# The Danish helicopter emergency medical service database: high quality data with great potential

**DOI:** 10.1186/s13049-019-0615-5

**Published:** 2019-04-05

**Authors:** Karen Alstrup, Jens Aage Kølsen Petersen, Charlotte Barfod, Lars Knudsen, Leif Rognås, Thea Palsgaard Møller

**Affiliations:** 1 Research and Development, Pre-hospital Emergency Medical Services, Central Denmark Region, Olof Palmes Allé 34, 8200 Aarhus N, Denmark; 2 Department of Anaesthesiology, Aarhus University Hospital, Aarhus, Denmark; 3 The Danish Air Ambulance, Aarhus, Denmark; 4 Emergency Medical Services, Copenhagen, Denmark

**Keywords:** Helicopter EMS, Data quality, Validity, Quality indicators

## Abstract

**Background:**

The Danish Helicopter Emergency Medical Service (HEMS) is part of the Danish pre-hospital response offering advanced patient care on scene and during rapid transport to definitive care.

Monitoring HEMS performance and the quality of critical care has high national as well as international priority underlining the need for research in this field.

The data quality of the Danish HEMS database is unknown. Furthermore, a set of quality indicators (QI) developed by an international collaboration group (EQUIPE) potentially for use in physician-staffed EMS, has recently been presented.

The aim of the current study was to present the design and data quality of the Danish helicopter database, and to evaluate the coverage of available variables in the database according to the QIs proposed.

**Method:**

The study included all helicopter dispatches between October 1st 2014 and April 30th 2018.

The database layout and data entering procedure, as well as the key variables and data completeness were described. Furthermore, missing data and misclassifications were addressed.

Lastly, the 26 QIs proposed by the EQUIPE-collaboration were evaluated for coverage in the HEMS database.

**Results:**

A total of 13,392 missions were included in the study. The database includes a broad spectrum of mission- and patient-specific data related to the pre-hospital pathway of acutely ill or injured patients in a national coverage. Missing data for the majority of variables is less than 6.5%. The percentage of completed report forms has increased over time and reached 99.9% in 2018.

Misclassification were observed for 294 patients in the study period corresponding to 3,7%.

Less than half of the QIs proposed by the EQUIPE-collaboration group were directly available from the database.

**Conclusions:**

Helicopter Emergency Medical Services in Denmark are a new and sparsely investigated health care provider. The database contains nearly all missions dispatched by the five regional Emergency Medical Dispatch Centres. Generally, the data quality is considered high with great potential for future research.

Potential quality indicators as proposed by the EQUIPE-collaboration group could inspire the configuration and design of the next version of Hemsfile creating an even more solid basis for research and quality improvement.

**Electronic supplementary material:**

The online version of this article (10.1186/s13049-019-0615-5) contains supplementary material, which is available to authorized users.

## Background and aim of the database

Pre-hospital care has evolved dramatically during the past decades from being a basic transport facility into offering advanced patient care on scene and during transportation.

In recent years, much focus has been placed on the utilisation and effect of pre-hospital resources underlining the need for research and system performance evaluations.

The Danish Helicopter Emergency Medical Service (HEMS) is part of the Danish pre-hospital emergency medical services (EMS). Following two trial periods between 2010 and 2014 with only one locally placed air ambulance [1, 2], HEMS became national on October 1st 2014 with implementation of three air ambulances placed to ensure geographical and timely coverage. HEMS is mainly dispatched for patients suspected to be suffering from time critical emergencies such as stroke, cardiac arrest, acute myocardial infarction, or severe trauma; conditions where appropriate and timely treatment on scene and fast transfer to specialised care is of outmost importance.

The use of HEMS in Denmark is on the increase [3]. Thus, the helicopters were dispatched 4199 times in 2017 compared with 3058 events in 2015. Monitoring EMS performance and the quality of critical care has high national as well as international priority [4, 5], and using quality measurements in the evaluating of delivered health care has become an integrated part of many organisations.

Although physician staffed EMS have long traditions and are well established in many countries, relatively little is known about their performance [6]. In a recent study, Haugland et al. presented a set of multidimensional quality indicators (QI) developed by an international expert group, the EQUIPE-collaboration group, potentially for use in physician-staffed EMS across borders. Twenty-six specific QIs were identified representing a broad approach to quality measurements [7].

The performance of the national HEMS in Denmark is largely unexplored. Guidelines for HEMS use are adjusted regularly, but currently, no validated or clearly defined quality measures exist.

The Danish HEMS database, *Hemsfile*, was established in 2010 along with the introduction of the first HEMS trial. The overall objective of the database is to monitor, assess and improve the quality of clinical care, as well as to create a basis for observational research and, in time, high quality randomised controlled trials. The database is approved by the Danish Data Protection Agency and administered by the Danish HEMS organisation.

The aim of the current study is to present the design and data quality of Hemsfile, and to evaluate the coverage of available variables in Hemsfile according to the QIs suggested by the EQUIPE-collaboration group.

## Methods

### Study population

The study population consists of all helicopter dispatches between October 1st, 2014 and April 30th, 2018, thereby including missions registered in the period with national HEMS.

### Setting

The Danish National Health Services provides free and universal tax-supported health care for every citizen, including pre- and in-hospital services as well as access to general practitioners.

Each of the five Danish regions has its own pre-hospital organisation (health trusts) with an emergency medical dispatch centre (EMDC) [8]. The pre-hospital organisations are responsible for the care and treatment on scene and during transportation until the patient reaches the hospital.

**Fig. 1:**
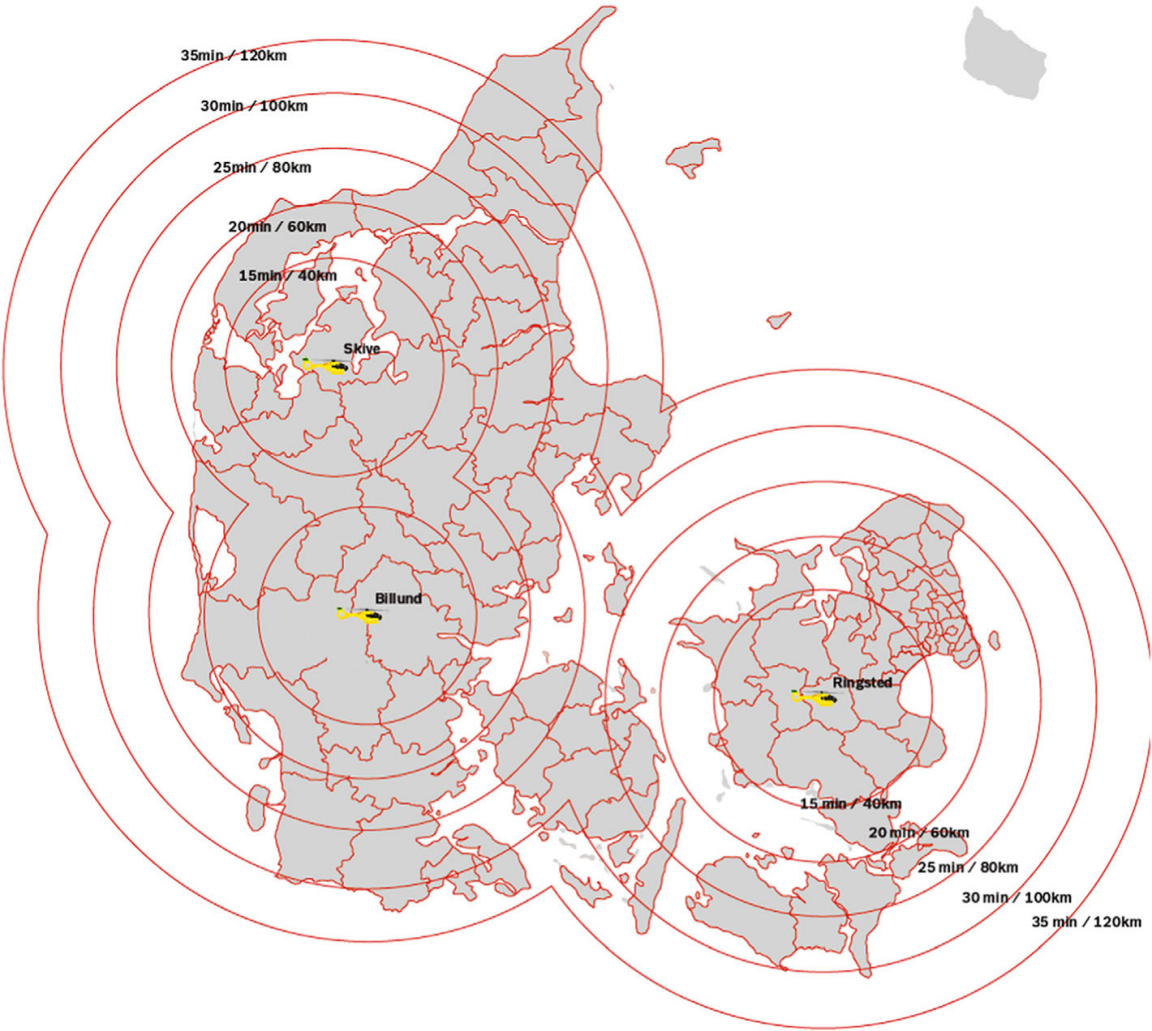
The location of the bases including reaching distances

HEMS acts as a supplement to ground EMS (ambulances and nurse- or physician-staffed rapid response vehicles). HEMS in Denmark is organised similar to many HEMS systems in Scandinavia and the Central Europe, staffed by a consultant-level anaesthesiologist, a pilot and a specially trained paramedic and operating 24 h/day, 7 days a week ([9–11] http://www.akutlaegehelikopter.dk). The helicopters are equipped for visual and instrumental flight conditions as well as night operations. Most parts of the country can be reached within 30 min (Fig. 1). The service is governmentally founded.

Helicopter dispatch is coordinated from the five EMDCs according to a specific HEMS dispatch guideline (http://www.akutlaegehelikopter.dk). The decision to dispatch a helicopter is taken by the medical dispatchers who are healthcare professionals (specially trained nurses, ambulance technicians and paramedics) handling medical emergency calls from the public dialling the emergency phone number 112. Technical dispatchers trained in logistics undertake the actual dispatch.

The Danish HEMS undertakes both primary critical care missions (request from citizens through emergency calls and crew request from ambulances and rapid response vehicles on scene) and time critical secondary missions (inter-facility transfers). Furthermore, the HEMS also provides pre-hospital care and transport for less ill or injured patients located on islands not connected by road to the mainland. Every HEMS dispatch is registered in Hemsfile within hours after the mission.

### Data registration and variables

The HEMS physicians (consultant-level anaesthesiologists) are responsible for data registration. On mission, they document operational and medical data in a paper-based patient data form, which is subsequently manually entered into the electronic Hemsfile. Based on the classification of the mission performed, separate report forms are filled in. Thus, there is one report form for patients treated on scene and carried to hospital by HEMS (registration form 1 (RF1)); one for patients attended to and treated, but handed over to the ground unit staff or left on scene (registration form 2 (RF2)); one for aborted missions (missions cancelled in-flight) (registration form 3 (RF3)); and one for rejected HEMS missions (no take-off) (registration form 4 (RF4)). In addition, phone requests not leading to a HEMS mission are registered separately. Consequently, two report forms represent HEMS missions with a patient encounter (RF1 and RF2), and two represent missions with no patient contact (RF3 and RF4). The four report forms each comprise a predefined set of mandatory variables. During autumn 2015, a visual warning indication system was integrated in Hemsfile aiming to reduce the number of incomplete report forms. Except for timestamps, each variable is entered in an exclusive multiple-choice or drop-down menu procedure. In addition to the predefined variables entered, space is left for individual notes as free text, e.g. a short medical report, adverse events and educational events.

The variables in Hemsfile reflect the operational and the medical part of the mission. The operational data include base, crew, dispatching region, date and timestamps, transfer mode, and reason for cancellation/rejected mission. The medical data include the civil registry number assigned each Danish citizen, the severity of the patient’s condition using the National Advisory Committee for Aeronautics (NACA) severity score [12], the interventions performed, and the pre-hospital diagnosis assigned by the HEMS physicians based on the international classification of diseases, 10th edition (ICD-10).

### Quality indicators

Quality indicators have been defined for many patient subgroups [13–15]. They are measurement tools aiding clinicians or organisations in documenting and monitoring delivered health care, thereby providing a basis for quality assessment, improvement and research. Optimally, they should rely on robust scientific evidence, but a structured consensus-based process may be used where scientific evidence is lacking [16].

The EQUIPE-collaboration group included 18 representatives from 8 nations with expertise in different areas related to EMS [7]. This expert panel reached consensus on QIs addressing 6 quality dimensions (timeliness, safety, efficiency, equity, effectiveness and patient-centeredness) as stated by the Institute of Medicine [17].

To cover these 6 quality dimensions, three groups of quality indicators described by Donabedian et al. [16] were used; *structure indicators* which relate to the setting or organisational infrastructure in which an event occur, *process indicators* which relate to the interaction with the patient, and *outcome indicators* which are related to the impact on the patient status.

## Results

**Fig. 2:**
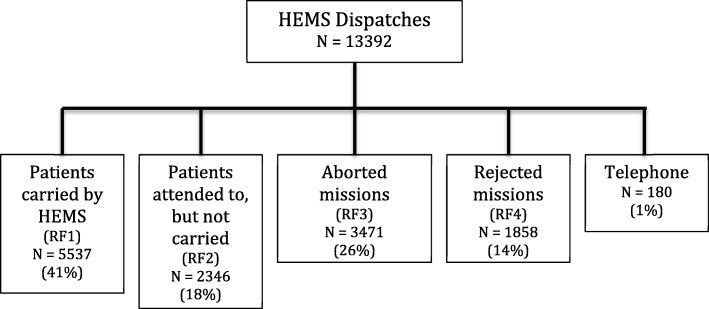
Distribution of HEMS missions based on the four report forms. Rejected missions reflect dispatches not leading to take off. Aborted missions are missions cancelled in-flight after take off

By the end of April 2018, the database contained 13,392 dispatches. The distribution of the dispatches, based on the four different report forms, is presented in Fig. 2.

For each mission with a patient encounter (RF1 and RF2), Hemsfile holds both operational and medical data. Aborted and rejected missions do not result in patient encounters, and subsequently medical data are not entered into the corresponding report forms (RF3 and RF4). Table 1 shows the overall variables available in each of the four forms.

**Table 1:** Variables available from the four different report forms

	Carried (RF1)	Attended to (RF2)	Aborted (RF3)	Rejected (RF4)
Variable				
CRS number	x	x		
Base	x	x	x	x
Crew	x	x	x	x
Dispatch region	x	x	x	x
Date	x	x	x	x
Time of alarm	x	x	x	x
Location	x	x	x	x
Timestamps	x	x	x	
NACA score	x	x		
Interventions	x	x		
ICD-10 diagnosis	x	x		
Transfer Mode	x	x		
Reason for cancellation			x	
Reason for rejected mission				x

The variables and a brief description of the content and the percentage of data completeness on each of the variables are presented in Table 2.

**Table 2:** Operational and medical variables, including content and completeness of each variable in Hemsfile

Variable	Content	Completeness (%)
Operational		
Hems ID	Unique number for identification of the event	100
Type of mission	Four separate report forms (patient treated and carried (RF1), patient attended to, but not carried (RF2), aborted missions (RF3), rejected missions (RF4)) and a telephone registration	100
HEMS base	Location of HEMS unit (Skive, Billund, Ringsted)	100
Crew		
Physician	Name of physician on call	100
Paramedic	Name of paramedic on call	98.6
Pilot	Name of pilot on call	98.6
Dispatching region	One of the five Danish regions (Capital Region, Region of Zealand, Central Denmark Region, Northern Region, Southern Region)	100
Date	Date of event	100
Timestamps		
Time of alarm	Defined as time of dispatch	100
Airborne	Defined as time of leaving the helipad at the base	97.6
Arrival on-scene	Defined as time of arrival on scene	99.9
Departure from the scene	Defined as time of helicopter take-off from the scene	99.0
Arrival at hospital	Defined as time of arrival at hospital	85.1
Patient management	Thirteen options (presented in Table 3)	100
Destination	Receiving university or regional hospital	100^a^
Island	Mission to an island not connected to the mainland (yes/no)	89.1^b^
Reason for aborted mission	Eight options (landing impossible, cancelled by RRV staff, cancelled by EMDC staff, cancelled by ambulance staff, redirected, weather, technic or other)	99.5
Reason for rejected mission	Eight options (duty-time, no need, no time gain, landing impossible, weather, concurrency, technic or other)	99.7
Medical		
Civil registry number	Unique 10-digit personal identifier	93.6^c^
NACA score	Used to assess the severity of illness/injury. Score ranges from 0 to 7	99.9
ICD-10	Pre-hospital diagnosis based on the International Classification of Diseases, 10th edition	99.8
Interventions		
Airway management	Three options (yes intubation by HEMS physician, yes intubation by RRV physician or no)	99.2
Ultrasound examination	Ultrasound examination by HEMS physician (yes/no)	98.2
Mechanical CPR (LUCAS)	Five options (yes at the scene, yes at the scene and during transfer, yes at the scene but not during transfer, yes stand-by during transfer or no)	96.2
Intraosseous access	Intraosseous access by HEMS physician (yes/no)	97.7
Thoracic intervention	Four options (yes thoracotomy, yes chest tube placement, yes thoracostomy or no)	95.8
Blood products	Red blood cells or plasma administered by HEMS physician (yes/no)	98,0

Abbreviations: *RRV* Rapid Response Vehicle, *NACA score* National Advisory Committee for Aeronautics score, *CPR* cardiopulmonary resuscitation, *NA* not available

^a^ Only patients carried by HEMS are included

^b^ The variable *Island* was added to the dataset in 2015

^c^ Per cent of valid civil registry numbers

### Data quality

#### Data reliability

As data registration is restricted to a limited number of physicians (twelve at each base) a uniform data collection is possible for most variables. However, variables related to e.g. patient status such as the NACA score and diagnostics are subject to personal interpretation and thus inter-rater variability. On-going education regarding correct interpretation and coding of variables contribute to improved data quality and uniformity.

**Fig. 3:**
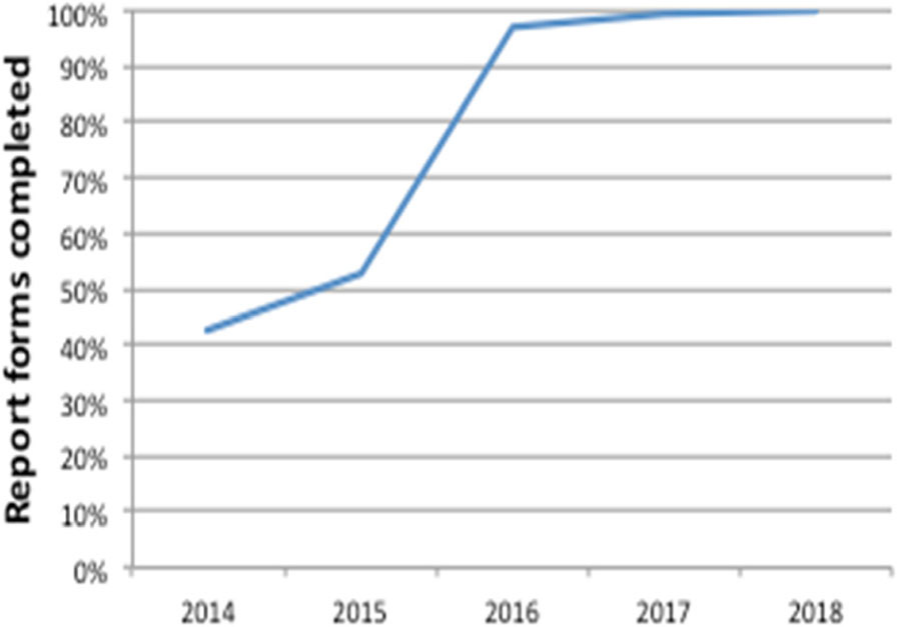
Report forms completed through the years since the implementation of the national HEMS

An incomplete report form is defined as a report form with one or more missing variables. The HEMS Medical Director continuously surveys and audits the data registrations, thereby reinforcing precise and complete registrations. The degree of completed report forms has increased over time and reached 99.9% in 2018 (Fig. 3).

#### Internal and external data validity

Hemsfile includes a broad spectrum of national mission- and patient-specific data related to the pre-hospital pathway of acutely ill or injured patients. Generally, the data completeness is considered high. Missing data is less than 6.5% for the majority of variables. Missing civil registry numbers are observed in 62 (0,8%) cases and incomplete civil registry number registrations are found in 439 (5,6%) cases (RF1 and RF2).

However, surveys of the dataset demonstrate several misclassifications. As illustrated in Table 3, twelve different options in the variable *patient management* exist when entering data in the two report forms RF1 and RF2. It appears that a patient who has been carried by HEMS to hospital may also have a registration as e.g. being transported by ambulance or escorted by Rapid Response Vehicle (RRV) staff, pronounced dead on scene or completed on scene, and patients attended to, but not carried by HEMS, may have been registered as being carried by HEMS. Misclassification was observed for 294 patients in the study period corresponding to 3,7% of the missions.

**Table 3:** Data entering options in the two report forms RF1 and RF2. The numbers indicate misclassified patients

	Carried (RF1)	Attended to (RF2)
Patient management		
Completed on scene	3	
Admitted to hospital by ambulance	41	
Admitted to hospital by paramedic/nurse		
Escorted by RRV	181	
Carried by HEMS		17
Inter facility transfer		
Escorted by HEMS physician in ambulance		41
Standy (fires, police requests	1	
Patient not found		
Patient dead on scene	1	
Patient pronounced dead on scene	7	
Patient inaccessible	2	

Also, timestamps represent a field with examples of imprecise registrations and subsequently extreme values probably due to the registration procedure. One hundred and ten missions (1,54%) had a response time higher than 60 min and 32 missions (0,45%) had a response time > 90 min (interhospital transfers excluded).

Regarding the external validity: the use of HEMS is restricted to patients suffering time critical conditions located in the most rural parts of the country, including islands, with long distances to specialised care. Thus, the HEMS population represents a selected group of patients and may not be comparable to the general unselected Danish pre-hospital mixed urban/rural population handled in e.g. physician-staffed ground EMS. However, our population might be comparable with other HEMS populations in settings almost similar to the Danish.

### Availability of the EMS quality indicators in Hemsfile

The EQUIPE-collaboration group identified a set of 26 QIs, 15 response-specific and 11 system-specific QIs, as potentially important measures in pre-hospital physician-led care. Most of the proposed response-specific QIs evaluate the care for the patient (process indicators). The system-specific QIs describe system or organisational characteristics and it is suggested by the group that these indicators are registered once a year.

Table 4 lists the QIs and their availability in Hemsfile.

**Table 4:**
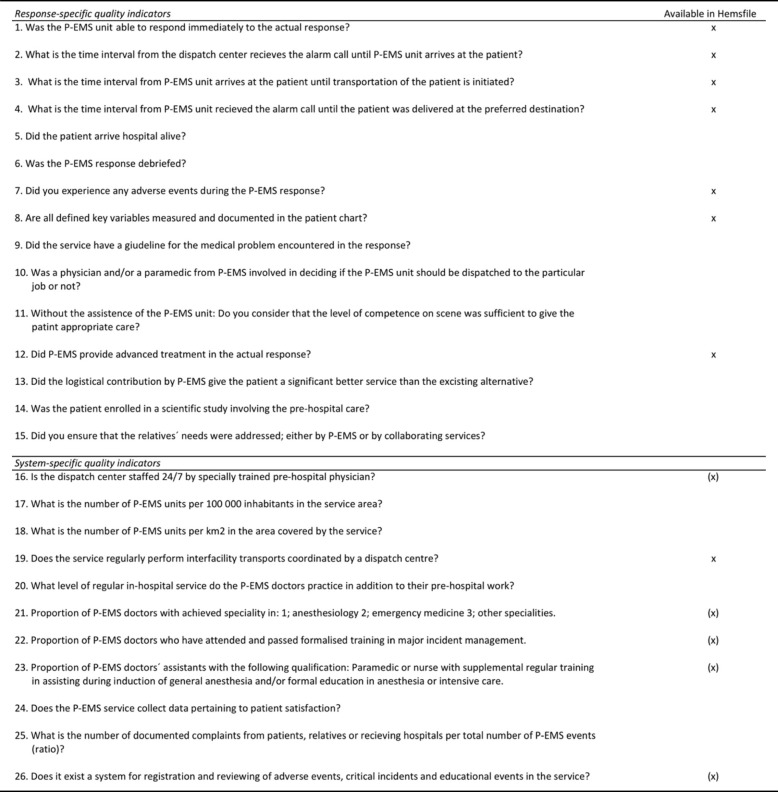
List of 26 quality indicators for physician-staffed emergency medical services (P-EMS) and their availability in Hemsfile

## Discussion

The objective of this study was to describe in detail the design and data quality of the Danish HEMS database, and to evaluate the coverage of the variables in Hemsfile according to the QIs proposed by the EQUIPE-collaboration group. The study included 13,392 missions registered in Hemsfile in the first 3 years and 7 months with national HEMS. We have no information on missions not dispatched from the EMDCs due to concurrencies or helicopters/crews being out of service.

To our knowledge it is the first study of its kind dealing with key topics for valid and reliable research. Assessment of data quality is important, and a prerequisite, when interpreting results from register-based studies. The overall data quality is considered high with a high degree of data completeness for the large majority of variables.

However, the study has limitations. Hemsfile includes four report forms each containing a specific set of variables mandatory for registration. Some variables, especially those related to patient status, are prone to inter-rater variability. Evaluating this topic is important but was not the focus of this study.

Moreover, missing or incomplete civil registry numbers was observed in 6,4%. This is in contrast to other pre-hospital or emergency studies reporting on missing values in up to 20% of the cases [18]. Further analyses are needed to assess if these patients represent a random selection of the population or can be explained by e.g. tourists without a Danish civil registry number. Thus, how inter-rater variability and missing data might affect or bias future study results are unknown, but must be addressed in future HEMS study designs.

It appears that by adding the visual warning indication system to Hemsfile the amount of patients with a complete report form increased dramatically. This is a simple way of improving data completeness, and when combined with on-going educational efforts, training and audits, the degree of completed reports reached almost 100% at the end of the study period. However, the layout of the database is not completely intuitive and clear. Multiple-choice menus allowing conflicting registrations, e.g. patient carried and completed on scene at the same time, may lead to misunderstandings and errors when used. Furthermore, manually entered data such as timestamps, which are first registered in a paper-based data form and subsequently entered into Hemsfile, are prone to registration errors. A random sample on 35 of the extreme values observed (response time > 60 min) showed obvious errors in the entering procedure suggesting that electronical data capturing could be beneficial. A well designed electronic data collection tool, intuitive and easy in use, not only increases the data quality by reducing the risk of errors and missing values and eliminating inconsistencies, but also saves time and ensures real-time data when integrated in the helicopters. The technological infrastructure of Hemsfile is presently under reconstruction. When completed, misclassifications and technical errors are likely to be reduced.

Comparing the timestamps registered by the physicians with the timestamps registered by the pilots could provide a more precise estimate of the response-times. This validation requires access to the aviation database, *NOLAS*, which we did not have. Timestamps have much attention and are a central aspect of EMS data collection, as these are well defined for each pre-hospital unit and easy to evaluate. Accordingly, they are often used as QIs. HEMS offer advanced care for patients suffering time-critical conditions where time to initiation of treatment as well as time to definitive care is considered important for the patient outcome. In these cases precise registration of timestamps is crucial in the evaluation of the service. However, not all patients carried by HEMS are suffering time-critical conditions and thus, timestamps do not always reflect quality of care and should not stand alone as single quality measurements.

About half of the proposed response-specific QIs are available from Hemsfile. Hemsfile was initially designed for mission reporting, but without specific reflections on QIs. The selection of variables was based on experiences from a previous pre-hospital database related to the Danish physician-staffed rapid response vehicles. Knowledge and quality assessments from then formed the design process of Hemsfile in 2009–10. The choice of proposed QIs are based on the latest knowledge in the field. This gap between the first introduction of Hemsfile and the development of QIs may explain the modest number of available QIs in Hemsfile. However, some of the lacking QIs are to be found elsewhere in our system, ex. QI 9 and QI 10.

The QI identification and implementation is an initial step towards monitoring quality. Using the experience from the process described by the EQUIPE-collaboration group forms a basis for further discussions on how and to which extent QIs should be implemented in a Danish setting. The pre-hospital field is characterised by patient heterogeneity and system complexity. Measuring the quality of (H)EMS is a challenging task, which is also underlined in the review by Sayed et al. [19], and a broad and wide-ranging approach may be preferable.

### Perspectives and future research

Data from Hemsfile has been used in several studies related to the trial periods (2010–2014) covering different topics, including studies on patient outcome, HEMS effectiveness and socio-economics for selected patient subgroups [1, 2, 20–22]. The period with national HEMS has been investigated sparsely [23].

Linkage of pre-hospital data with other national public registries and databases through the civil registry number assigned each Danish citizen [24, 25], offers unique opportunities for research regarding follow-up and healthcare management.

Variables concerning the clinical status of the patients such as severity of illness/injury, pre-hospital diagnostics and pre-hospital interventions performed are characterized by a high degree of data completeness (95.8–99.9%). Further evaluation of these performance indicators may contribute in the assessment of dispatch accuracy, which is a key aspect in the overall evaluation of HEMS.

Increasing our knowledge of the Danish HEMS patient population and the HEMS mission profile is fundamental to improve not only dispatch and resource utilisation, but also patient safety and patient outcome.

Collecting data and being able to compare them with data from other services, e.g. through a set of consensus-based QIs covering the pre-hospital patient pathway, is considered an important step in adding a valuable aspect into performance evaluation, research and collaboration. Therefore, providing an insight into our data source is essential for the purpose of facilitating comparison of services nationally as well as across borders in the future.

## Conclusion

The Helicopter Emergency Medical Service in Denmark is a new and sparsely investigated health care provider. The Hemsfile database is an important data source for research and quality improvement in relation to pre-hospital critical care. The database contains nearly all missions dispatched by the five regional Emergency Medical Dispatch Centres. Generally, the data quality is high with great potential for future research.

Potential quality indicators as proposed by the EQUIPE-collaboration group implemented in Hemsfile might create an even more solid basis for research and quality improvement inter-organisationally.

## Additional file

Additional file 1: Report form reflecting a carried patient. (DOCX 402 kb)

## Abbreviations

CPR: Cardiopulmonary resuscitation
EMDC: Emergency Medical Dispatch Centre
HEMS: Helicopter Emergency Medical Services
ICD-10: International Classification of Diseases, version 10
LUCAS: Mechanical cardiopulmonary resuscitation
NACA: National Advisory Committee for Aeronautics score
QI: Quality Indicators
RRV: Rapid Response Vehicle
