# Supplementary figures and images for: The Danish helicopter emergency medical service database: high quality data with great potential
Source: Scand J Trauma Resusc Emerg Med. 2019 Apr 5;27:38. doi: 10.1186/s13049-019-0615-5 (PMC6451291; doi:10.1186/s13049-019-0615-5)

Additional Material: Report form reflecting a carried patient.


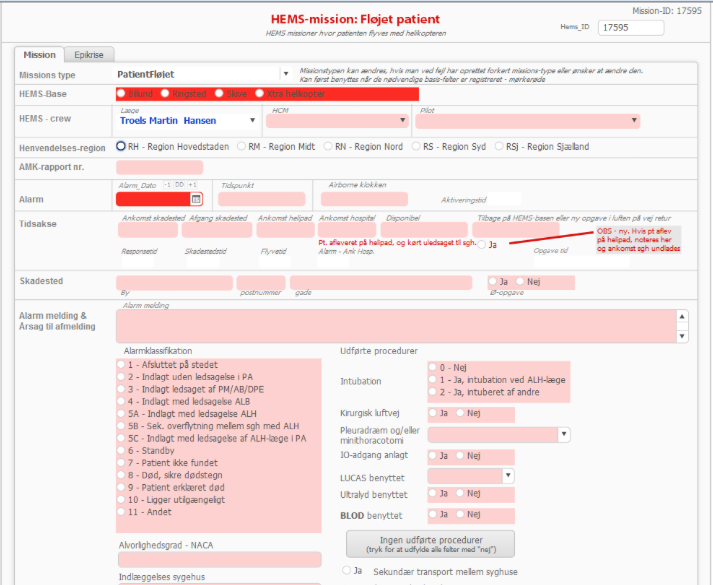


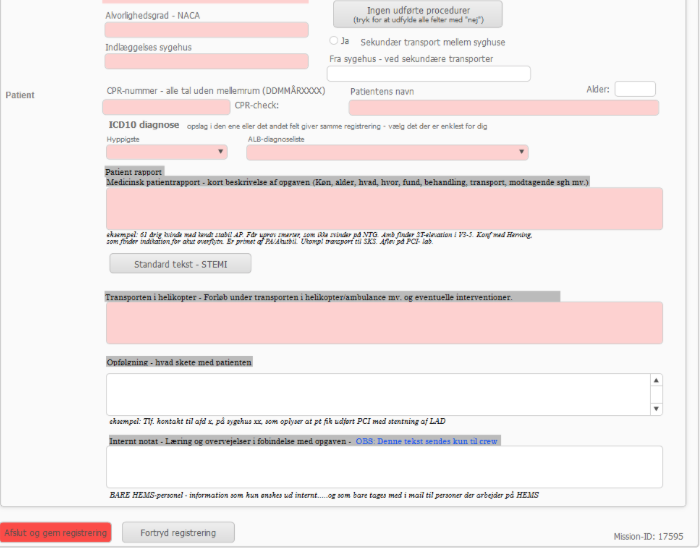

Supplement: Supplementary file 1 — Report form reflecting a carried patient. (DOCX 402 kb) [file 13049_2019_615_MOESM1_ESM.docx]
